# Random Forest for Predicting Treatment Response to Radioiodine and Thyrotropin Suppression Therapy in Patients With Differentiated Thyroid Cancer But Without Structural Disease

**DOI:** 10.1093/oncolo/oyad252

**Published:** 2023-09-05

**Authors:** Ri Sa, Taiyu Yang, Zexu Zhang, Feng Guan

**Affiliations:** Department of Nuclear Medicine, The First Hospital of Jilin University, Changchun, People’s Republic of China; Department of Nuclear Medicine, The First Hospital of Jilin University, Changchun, People’s Republic of China; Department of Nuclear Medicine, The First Hospital of Jilin University, Changchun, People’s Republic of China; Department of Nuclear Medicine, The First Hospital of Jilin University, Changchun, People’s Republic of China

**Keywords:** DTC without structural disease, ^131^I therapy, TSH suppression therapy, thyroglobulin, random forest

## Abstract

**Background:**

We aimed to develop a machine-learning model for predicting treatment response to radioiodine (^131^I) therapy and thyrotropin (TSH) suppression therapy in patients with differentiated thyroid cancer (DTC) but without structural disease, based on pre-treatment information.

**Patients and Methods:**

Overall, 597 and 326 patients with DTC but without structural disease were randomly assigned to “training” cohorts for predicting treatment response to ^131^I therapy and TSH suppression therapy, respectively. Six supervised algorithms, including Logistic Regression, Support Vector Machine, Random Forest (RF), Neural Networks, Adaptive Boosting, and Gradient Boost, were used to predict effective response (ER) to ^131^I therapy and biochemical remission (BR) to TSH suppression therapy.

**Results:**

Stimulated and suppressed thyroglobulin (Tg) and radioiodine uptake before the current course of ^131^I therapy were mostly attributed to ER to ^131^I therapy, while thyroid remnant available on the post-therapeutic whole-body scan at the last course of ^131^I therapy and TSH were greatly contributed to Tg decline under TSH suppression therapy. RF showed the best performance among all models. The accuracy and area under the receiver operating characteristic curve (AUC) for segregating ER from non-ER during ^131^I therapy with RF were 81.3% and 0.896, respectively. The accuracy and AUC for predicting BR to TSH suppression therapy with RF were 78.7% and 0.857, respectively.

**Conclusion:**

This study demonstrates that machine learning models, especially the RF algorithm are useful tools that may predict treatment response to ^131^I therapy and TSH suppression therapy in DTC patients without structural disease based on pre-treatment routine clinical variables and biochemical markers.

## Teaser

This article reports a machine learning model for predicting treatment response to radioiodine therapy and thyrotropin suppression therapy in patients with differentiated thyroid cancer but without structural disease, based on pre-treatment information.

**Implications for Practice**

The treatment of differentiated thyroid cancer (DTC) involves a combination of surgical intervention, radioiodine (^131^I) therapy, and thyrotropin (TSH) suppression therapy. The pre-treatment clinical variables and biochemical markers from patients may be useful for predicting treatment response to ^131^I therapy and TSH suppression therapy before treatment. Our study found that stimulated and suppressed thyroglobulin (Tg), and radioiodine uptake before therapy were mostly attributed to excellent response to ^131^I therapy, while thyroid remnant available on the post-therapeutic whole-body scan at the last course of ^131^I therapy and TSH were greatly associated with Tg decline under TSH suppression therapy. Integrating these data into a random forest model may facilitate predicting treatment response to ^131^I therapy and TSH suppression therapy in patients with DTC but without structural disease.

## Introduction

Differentiated thyroid cancer (DTC) is the most common form of thyroid cancer, accounting for approximately 90% of all thyroid cancers.^1^ Due to the widespread of general health examinations, the majority of patients with DTC are diagnosed at an early stage, often without evidence of persistent disease following total thyroidectomy on imaging.^2^ Overall, the treatment of DTC involves a combination of surgical intervention, radioiodine (^131^I) therapy, and thyrotropin (TSH) suppression therapy. ^131^I therapy following surgery is used to destroy residual presumably benign thyroid tissue, and/or suspected but not identified remaining disease or biochemical evidence of persistent disease.^3,4^ Levothyroxine (LT4) is required for patients to substitute new-onsets thyroid hormone deficiency and suppress the malignancy spreading.^5^ A graded tailored approach of TSH suppression therapy has been proposed by ATA guidelines. This approach aims to maximize treatment benefit and decrease unnecessary adverse effects from thyroid hormone overtreatment.^6^

Thyroglobulin (Tg) level is commonly measured to assess the treatment effect and to monitor the recurrence or metastasis of disease after total thyroidectomy and ^131^I therapy.^7-10^ It is well accepted that either the stimulated Tg (Tg_off_) or the suppressed Tg (Tg_on_) is essential for disease management in patients with DTC but without structural disease.^11^ A low level of Tg following thyroid remnant means no active tumor in the body, and the time interval between serum Tg measurements can be lengthened to at least 12-24 months for these patients.^3^ By contrast, if a high level of Tg or a rise in Tg levels following thyroid remnant are detected, further imaging or other diagnostic tests may be necessary to determine the cause of the increase. Even if these patients had no evidence of structural disease, they are supposed to receive adjuvant therapy to reduce risk of recurrence or metastasis.^12^ Once Tg level drops to its nadir point, the initial risk of recurrence should be modified and the patient may be reclassified as having a subsequent very low risk of recurrence.^13^

While both ^131^I therapy and TSH suppression therapy are considered as effective approach for controlling Tg expression to a certain level in patients with DTC but without structural disease,^14,15^ there is still considerable controversy surrounding the Tg decline from ^131^I therapy or TSH suppressions therapy.^3,16,17^ Under this scheme, it is important to screen out benefit patients from non-benefit populations in the treatment of ^131^I therapy or TSH suppressions therapy. Aiming to maximize treatment effects of ^131^I therapy or TSH suppressions therapy, several previous studies have found out the influential factors that associated with treatment response to ^131^I therapy and TSH suppression therapy. However, the influential factors were mainly identified by univariate and multivariate analysis. The common statistical methods are ill-suited for handling complex information because it is difficult to capture the complexity of the internal relationship of these factors^18^; until recently, this has been a major limitation that prevents predicting treatment response to ^131^I therapy and TSH suppression therapy from pre-treatment clinical variables and biochemical markers.

The machine learning approaches “learn” potential patterns from past examples and detect difficult-to-recognize patterns from complex combinations of multiple meaningful factors.^19^ The use of machine learning models may provide additional insight into the complex relationships between these factors and treatment response particularly in cases where multiple factors interact to influence treatment response. We, therefore, reviewed a large series of patients with DTC but without structural disease treated by means of ^131^I therapy, subsequent TSH suppression therapy and followed up for a long time at a single institution, in order to establish and validate an artificial intelligence approach for predicting treatment response to ^131^I therapy and subsequent TSH suppression therapy in these entity by integrating the related pre-treatment routinely obtained factors that associated with curative effect in decision-making.

## Materials and Methods

### Study Populations

We retrospectively enrolled patients with DTC but without structural disease who underwent totally or nearly totally thyroidectomy with neck lymph node dissection between January 2011 and December 2020 in our institution. Patients were recruited for our study if (1) they received at least one course of ^131^I therapy; (2) they were over 18 years old; (3) they underwent TSH, Tg, antithyroglobulin antibody (TgAb), radioiodine uptake (RAIU%) test, ultrasound (US), and chest computed tomography (CT) before the current course of ^131^I therapy, 4-6 months after the last course of ^131^I therapy, and 12-14 months after the last course of ^131^I therapy. Patients were excluded with any of the following if (1) they demonstrated undetectable Tg_on_ (Tg_on_ < 0.2 ng/mL) before ^131^I therapy, (2) they had evidence of lymph node metastasis or distant metastasis within 6 months after ^131^I therapy, (3) the follow-up was incomplete. This study was approved by the Ethics Committee of the First Hospital of Jilin University.

An activity of 1.85-3.70 GBq (50-100 mCi) of ^131^I was orally administered for each course of ^131^I therapy. Three days later, a planar post-therapeutic whole-body scan (Rx-WBS) was performed with SPECT/CT. The possible modulation of LT4 dosage was done at 1-6 months after ^131^I therapy. The assessment of therapeutic response to ^131^I therapy was revealed 4-6 months after the last course of ^131^I therapy.

To determine the independent factors for treatment response to ^131^I therapy in patients with DTC but without structural disease, the following pre-treatment factors were examined: age, sex, pathology, TNM-T, TNM-N, stage, risk stratification, TSH before Levothyroxine withdrawal (THW) (TSH_on_), Tg_on_, TgAb before THW (TgAb_on_), TSH at 4 weeks post-THW (TSH_off_), Tg_off_, TgAb at 4 weeks post THW (TgAb_off_), RAIU% before the current course of ^131^I therapy, and courses of ^131^I therapy before the current course of ^131^I therapy. While to determine independent factors for treatment response to TSH suppression therapy, the following factors were examined: age, sex, pathology, TNM-T, TNM-N, stage, risk stratification, RAIU% before the last course of ^131^I therapy, courses of ^131^I therapy, thyroid remnant on Rx-WBS after the last course of ^131^I therapy, TSH, Tg_on_, TgAb at 4-6 months post the last course of ^131^I therapy (TSH_six_, Tg_six_, TgAb_six_) were examined.

### Treatment Response to ^131^I Therapy

**Figure 1.**
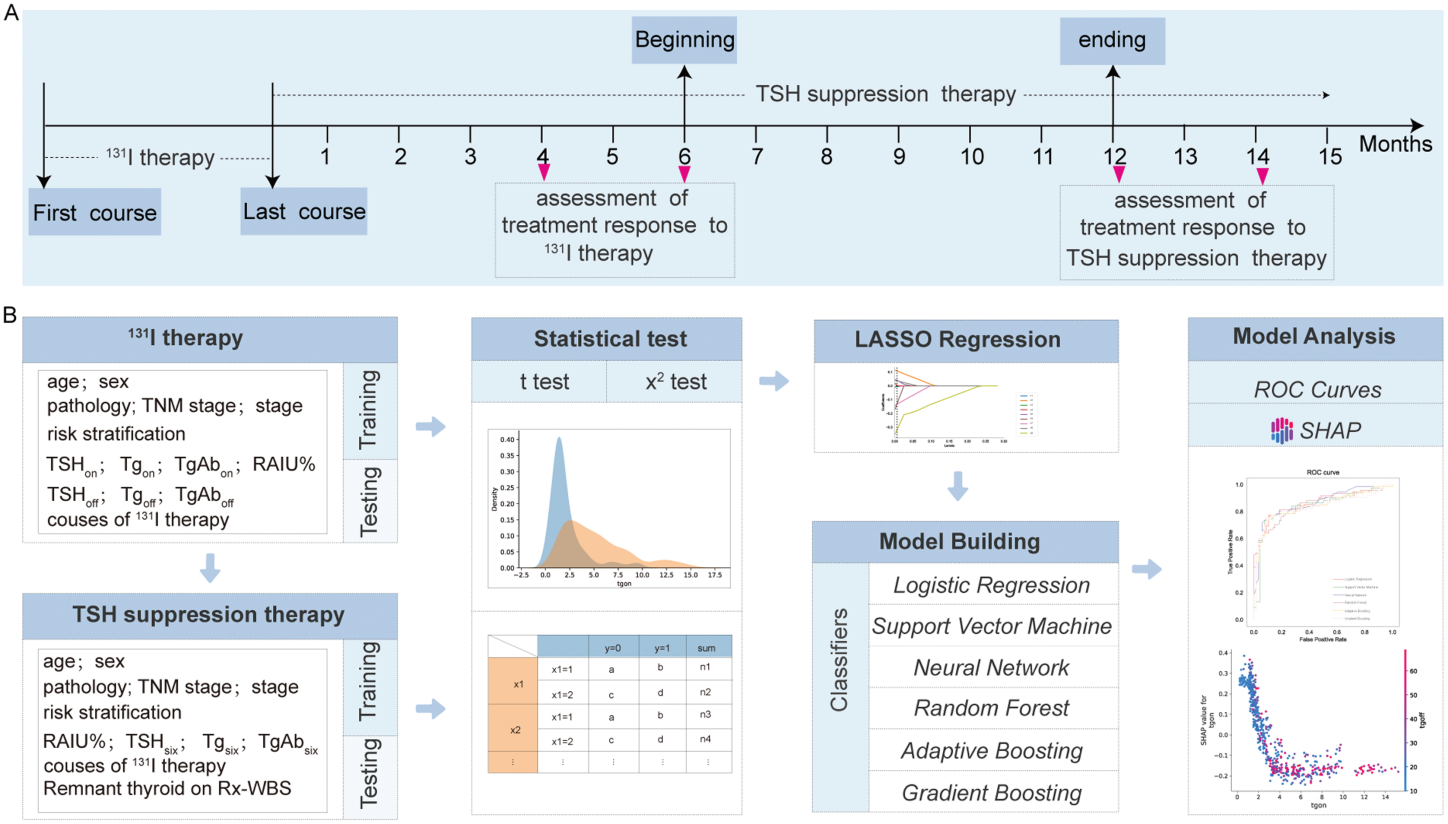
Workflow for predicting treatment response to radioiodine (^131^I) therapy and thyrotropin (TSH) suppression therapy in differentiated thyroid cancer patients without structural disease. (**A**) The time point of treatment response assessment in ^131^I therapy and TSH suppression therapy. (**B**) Workflow for establishment and validation of predicting models with different algorithms.

For treatment response to ^131^I therapy, the assessment was based on the ATA guidelines with minor modification, and it was conducted 4-6 months after the last course of ^131^I therapy. The time point of treatment response assessment is shown in Fig. 1A. Effective response (ER) to ^131^I therapy in patients with DTC but without structural disease is defined as undetectable Tg_on_ (< 0.2 ng/mL) while non-ER is defined as detectable Tg_on_ (≥ 0.2 ng/mL) or rising TgAb_on_ levels.

### Treatment Response to TSH Suppression Therapy

For treatment response to TSH suppression therapy, the assessment was based on the change rate of Tg_on_ level (△Tg_on_%), comparing Tg_on_ level at 6 months post the last course of ^131^I therapy (pre-TSH suppression therapeutic Tg_on_) with Tg_on_ level at 12-14 months post the last course of ^131^I therapy (post-TSH suppression therapeutic Tg_on_).The time point of treatment response assessment was shown in Fig. 1A. △Tg_on_% is defined as follows: (pre-therapeutic Tg_on_ level － post-therapeutic Tg_on_ level)/pre-therapeutic Tg_on_ level × 100%. In determining the categorization of biochemical response to TSH suppression therapy, the following standards were used: △Tg_on_% ≥ 25.0% indicated biochemical remission (BR), reflecting a decrease of Tg_on_ level by at least 25.0%, while △Tg_on_% < 25.0% meant non-BR, reflecting a rise of Tg_on_ level or the decrease of Tg_on_ level by <25.0%.

### Establishment and Evaluation of Machine Learning Algorithms

The study design flowchart is shown in Fig. 1B. The models were built and trained on a per person basis. All enrolled patients were randomly divided into a training cohort and a testing cohort with a ratio of 7:3 by 10 times cross-testing repeat. To identify the influential factors, continuous variables were analyzed using the Wilcoxon test, and categorical variables were analyzed using Chi-square test or Fisher’s exact test in the training cohort for initial screening. Factors with statistical significance (*P* < .05) were then dimensionality reduced using least absolute shrinkage and selection operator (LASSO) regression through 10 cross-testing. The dimensionality-reduced factors were used to build artificial intelligence approach. Six different machine learning algorithms including Logistic Regression (LG), Support Vector Machine (SVM), Random Forest (RF), Neural Networks (NN), Adaptive Boosting (ADA), and Gradient Boost (GB), were applied to model construction in the training cohort.

In order to evaluate the contribution of influential factors that made a predicted value in machine learning algorithm, SHapley Additive exPlanations (SHAP) summary plot was used. In the prediction of treatment response to ^131^I therapy, “0” was considered as ER, and “1” was defined as non-ER in the SHAP summary plot. Positive SHAP values increase the likelihood of prediction of non-ER to ^131^I therapy, whereas negative SHAP values decrease the likelihood of prediction of non-ER to ^131^I therapy. Similarly, in the prediction of treatment response to TSH suppression therapy, “0” was considered as BR, and “1” was defined as non-BR in SHAP summary plot. Positive SHAP values increase the likelihood of prediction of non-BR to TSH suppression therapy, whereas negative SHAP values decrease the likelihood of prediction of non-BR to TSH suppression therapy. SHAP summary plots allow for a visual interpretation of the model’s prediction and help to identify the factors that have the greatest impact on the prediction.

For all algorithms, model performance was primarily evaluated by the receiver operating characteristic (ROC) curve and the area under the ROC curve (AUC) in the testing cohort.

## Results

### Patient Characteristics for Predicting Treatment Response to ^131^I Therapy

Initially, a total of 843 patients without structural disease were enrolled for the training cohort. After the exclusion of 246 patients (89 patients demonstrated undetectable Tg_on_, and 56 patients had evidence of structural disease within 6 months after ^131^I therapy, and 101 patients with incomplete follow-up), 70.8% (597/843) patients were eligible for the training cohort. 339 patients were enrolled for the testing cohort. A total of 339 patients without structural disease were enrolled for the testing cohort. After the exclusion of 82 patients (32 patients with undetectable Tg_on_, and 8 patients with evidence of structural disease within 6 months after ^131^I therapy, and 42 patients with incomplete follow-up), 75.8% (257/339) patients were eligible for the testing cohort. Table 1 illustrates that all factors demonstrated no significant difference between training and testing cohort.

**Table 1.** Baseline characteristics of differentiated thyroid cancer patients without structural disease in the training and testing cohorts for prediction of treatment response to ^131^I therapy (*N* = 854).

Characteristic	Training cohort (*n* = 597)	Testing cohort (*n* = 257)	*P*-value
Sex			.25
Female	426 (71.4%)	173 (67.3%)	
Male	171 (28.6%)	84 (32.7%)	
Age, median (years)	45 (IQR, 36~54)	43 (IQR, 33~53)	.12
Pathology			1.00
PTC	586 (98.2%)	253 (98.4%)	
FTC	11 (1.8%)	4 (1.6%)	
TNM-T			.55
T1	397 (66.5%)	167 (65.0%)	
T2	98 (16.4%)	52 (20.3%)	
T3	66 (11.1%)	24 (9.3%)	
T4	36 (6.0%)	14 (5.4%)	
TNM-N			.16
N0	48 (8.1%)	14 (5.4%)	
N1a	215 (36.0%)	108 (42.0%)	
N1b	334 (55.9%)	135 (52.6%)	
Stage			.78
I	471 (78.9%)	208 (80.9%)	
II	122 (20.4%)	48 (18.7%)	
III	4 (0.7%)	1 (0.4%)	
IV	0 (0%)	0 (0%)	
Risk stratification			.87
Low	60 (10.1%)	25 (9.7%)	
Mediate	483 (80.9%)	206 (80.2%)	
High	54 (9.0%)	26 (10.1%)	
RAIU%, median	6.5 (IQR, 4.2~8.6)	6.0 (IQR, 3.7-8.5)	.12
TSH_on_, median (mIU/L)	0.32 (IQR, 0.08-0.75)	0.25 (IQR, 0.07-0.68)	.16
Tg_on_, median (ng/mL)	2.68 (IQR, 1.52-5.43)	2.61 (IQR,1.46-4.65)	.25
TgAb_on_, median (IU/mL)	11.70 (IQR, 10.00-17.09)	11.20 (IQR, 10.00-15.92)	.42
TSH_off_, median (mIU/L)	100.00 (IQR, 95.20-100.00)	100.00 (IQR, 95.40-100.00)	.23
Tg_off_, median (ng/mL)	22.09 (IQR, 15.37-36.75)	21.39 (IQR, 14.07-36.24)	.18
TgAb_off_, median (IU/mL)	11.87 (IQR, 10.00-17.80)	12.00 (IQR, 10.00-16.92)	.94
Courses of ^131^I therapy			.94
0	411 (68.8%)	178 (69.3%)	
≥1	186 (31.2%)	79 (30.7%)	

Abbreviations: PTC, papillary thyroid cancer; FTC, follicular thyroid cancer; TSH_on_, thyroid-stimulating hormone before Levothyroxine withdrawal; Tg_on_, suppressed thyroglobulin; TgAb_on_, antithyroglobulin antibody before Levothyroxine withdrawal; TSH_off_, thyroid-stimulating hormone after Levothyroxine withdrawal; Tg_off_, stimulated thyroglobulin; TgAb_off_, antithyroglobulin antibody after Levothyroxine withdrawal; IQR, interquartile range; RAIU, radioiodine uptake.

### Predicting Treatment Response to ^131^I Therapy

As shown in Table 2, 45.2% (270/597) patients received ER to ^131^I therapy, and 54.8% (327/597) patients obtained non-ER to ^131^I therapy in the training cohort, while 45.5% (117/257) patients received ER to ^131^I therapy, 54.5% (140/257) patients obtained non-ER to ^131^I therapy in the testing cohort.

**Table 2.** Comparison of clinical characteristics of differentiated thyroid cancer patients without structural disease who obtained ER or non-ER to ^131^I therapy in the training and testing cohorts (*N* = 854).

Characteristic	Training cohort (*n* = 597)			Testing cohort (*n* = 257)		
	ER (*n* = 270)	Non-ER (*n* = 327)	*P*-value	ER (*n* = 117)	Non-ER (*n* = 140)	*P*-value
Sex			.03			.18
Female	205 (75.9%)	221 (67.6%)		84 (71.8%)	89 (63.6%)	
Male	65 (24.1%)	106 (32.4%)		33 (28.2%)	51 (36.4%)	
Age, median (years)	45 (IQR, 36-53)	45 (IQR, 36-54)	.69	44 (IQR, 35-55)	42 (IQR, 32-52)	.07
Pathology			1.00			.13
PTC	265 (98.1%)	321 (98.2%)		117 (100.0%)	136 (97.1%)	
FTC	5 (1.9%)	6 (1.8%)		0 (0.0%)	4 (2.9%)	
TNM-T			.69			.64
T1	184 (68.1%)	213 (65.1%)		77 (65.8%)	90 (64.3%)	
T2	39 (14.4%)	59 (18.0%)		25 (21.4%)	27 (19.3%)	
T3	30 (11.1%)	36 (11.0%)		8 (6.8%)	16 (11.4%)	
T4	17 (6.3%)	19 (5.8%)		7 (6.0%)	7 (5.0%)	
TNM-N			.01			.46
N0	24 (8.9%)	24 (7.3%)		8 (6.8%)	6 (4.3%)	
N1a	116 (43.0%)	99 (30.3 %)		52 (44.4%)	56 (40.0%)	
N1b	130 (48.1%)	204 (62.4%)		57 (48.8%)	78 (55.7%)	
Stage			.69			.09
I	216 (80.0%)	255 (78.0%)		89 (76.0%)	119 (85.0%)	
II	53 (19.6%)	69 (21.1%)		27 (23.1%)	21 (15.0%)	
III	1 (0.4%)	3 (0.9%)		1 (0.9%)	0 (0%)	
IV	0 (0%)	0 (0%)		0 (0%)	0 (0%)	
Risk stratification			.01			.01
Low	36 (13.3%)	24 (7.3%)		16 (13.6%)	9 (6.4%)	
Mediate	224 (83.0%)	259 (79.2%)		96 (82.1%)	110 (78.6%)	
High	10 (3.7%)	44 (13.5%)		5 (4.3%)	21 (15.0%)	
RAIU%, median	6.9 (IQR, 4.9-8.7)	6.1 (IQR, 3.5-8.5)	.01	5.9 (IQR, 3.8-8.4)	6.0 (IQR, 3.7-8.7)	.96
TSH_on_, median (mIU/L)	0.39 (IQR, 0.09- 0.83)	0.27 (IQR, 0.07-0.70)	.03	0.22 (IQR, 0.07-0.57)	0.28 (IQR, 0.08-0.71)	.14
Tg_on_, median (ng/mL)	1.60 (IQR, 1.24-2.44)	4.39 (IQR, 2.52-7.07)	.01	1.43 (IQR, 1.16-2.23)	4.32 (IQR, 2.51-6.51)	.01
TgAb_on_, median (IU/mL)	11.62 (IQR, 10.00-17.31)	11.73 (IQR, 10.00-17.05)	.87	11.6 (IQR, 10.00-16.09)	10.71 (IQR, 10.00-15.65)	.13
TSH_off_, median (mIU/L)	100.00 (IQR, 97.70-100.00)	100.00 (IQR, 88.10-100.00)	.13	100.00 (IQR, 92.70-100.00)	100.00 (IQR, 96.66-100.00)	.58
Tg_off_, median (ng/mL)	16.37 (IQR, 12.00-22.60)	30.83 (IQR, 21.00-47.00)	.01	15.10 (IQR, 11.97-19.67)	31.89 (IQR, 20.29-42.98)	.01
TgAb_off_, median (IU/mL)	11.43 (IQR, 10.00-16.70)	12.00 (IQR, 10.00-18.20)	.27	11.50 (IQR, 10.00-15.69)	12.70 (IQR, 10.00-17.39)	.14
Courses of ^131^I therapy			.01			.01
0	221 (81.9%)	190 (58.1%)		96 (82.1%)	82 (58.6%)	
≥1	49 (18.1%)	137 (41.9%)		21 (17.9%)	58 (41.4%)	

Abbreviations: ER, excellent response; PTC, papillary thyroid cancer; FTC, follicular thyroid cancer; TSH_on_, thyroid-stimulating hormone before Levothyroxine withdrawal; Tg_on_, suppressed thyroglobulin; TgAb_on_, antithyroglobulin antibody before Levothyroxine withdrawal; TSH_off_, thyroid-stimulating hormone after Levothyroxine withdrawal; Tg_off_, stimulated thyroglobulin; TgAb_off_, antithyroglobulin antibody after Levothyroxine withdrawal; IQR, interquartile range; RAIU, radioiodine uptake.

**Figure 2.**
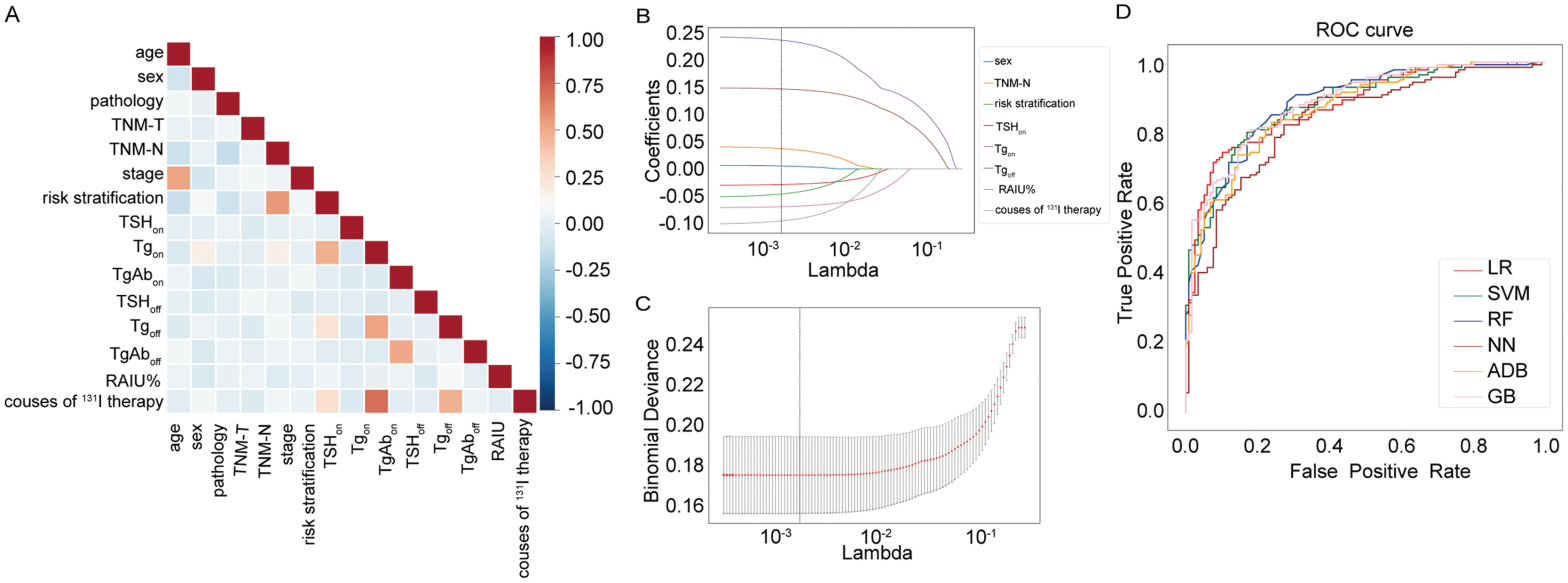
Establishment and validation of machine learning model for predicting treatment response to radioiodine (^131^I) therapy in differentiated thyroid cancer patients without structural disease. (**A**) Internal correlation between the enrolled factors associated with ^131^I therapy. (**B**) The least absolute shrinkage and selection operator for influential factors that associated with treatment response to ^131^I therapy. (**C**) The optimal value of λ at the minimum mean square error for feature selection. (**D**) Area under the receiver operating characteristic curve for predicting treatment response to ^131^I therapy.

There was no high internal correlation between the factors, as shown in Fig. 2A. Several variables including sex, TNM-N, risk stratification, RAIU%, Tg_on_, TSH_off_, Tg_off_, and courses of ^131^I therapy before the current course of ^131^I therapy showed significant difference in DTC patients with and without ER to ^131^I therapy after initial screening and LASSO regression . These factors were selected as the best predictors for the probability of non-ER to ^131^I therapy, as shown in Fig. 2B and 2C.

LG, SVM, RF, NN, ADA, and GB were used to build multiple models during the training process, and the performance of each algorithm was evaluated by AUC in the testing cohort. The results showed that these different algorithms had comparable performance in the prediction of treatment response to ^131^I therapy, with RF having the best accuracy of 81.3% and AUC of 0.896, as shown in Fig. 2D and Supplementary Table S1.

**Figure 3.**
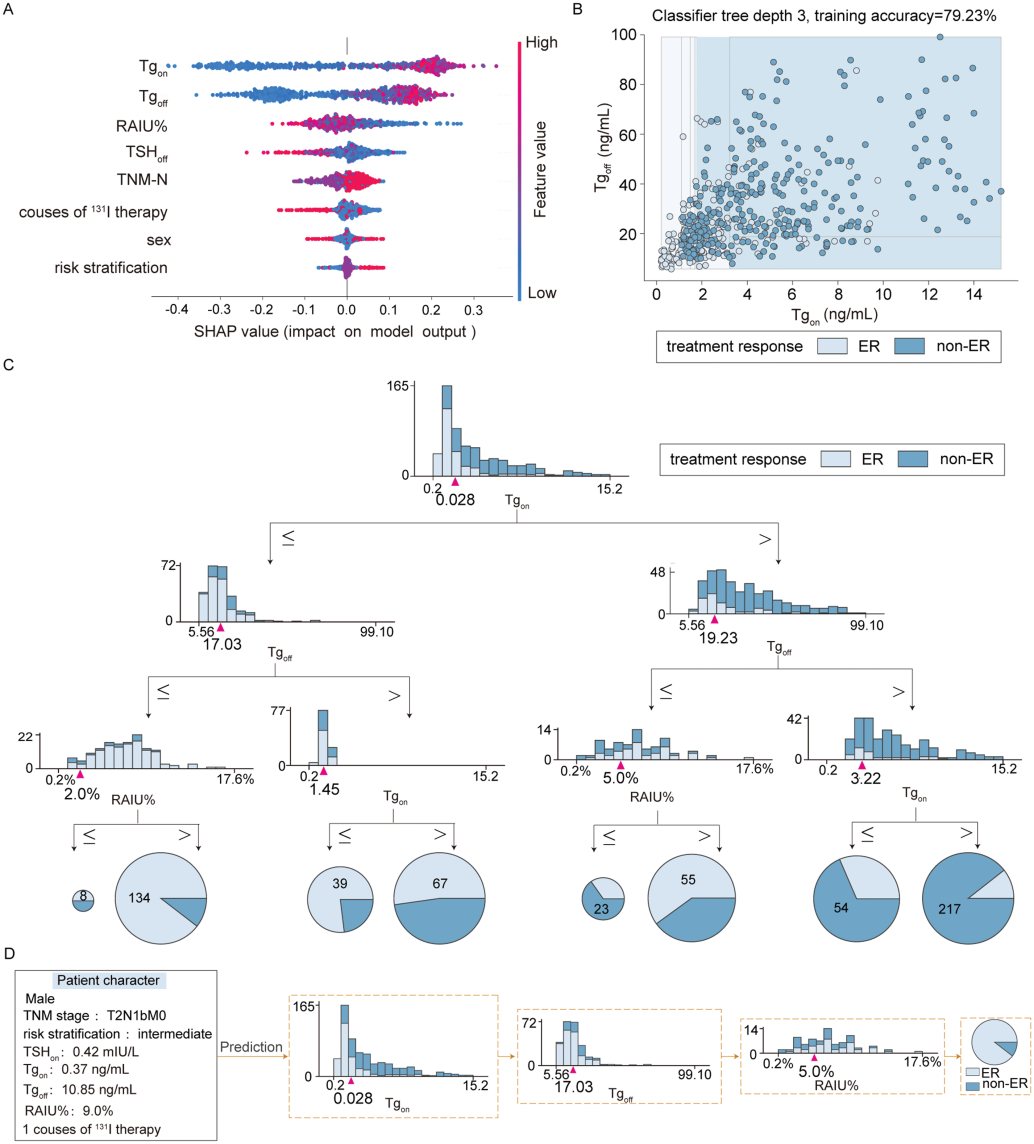
Random forest (RF) model for predicting treatment response to radioiodine (^131^I) therapy in differentiated thyroid cancer patients without structural disease. (**A**) SHapley Additive exPlanations summary plot for influential factors. (**B**) Correlation between stimulated and suppressed thyroglobulin in RF. (**C**) One of the RF ensembles of tree for predicting treatment response to ^131^I therapy. (**D**) A random sample of a patient using RF to predict treatment response to ^131^I therapy.

The role of all influential factors in RF is demonstrated by SHAP analysis in Fig. 3A. Higher levels of TNM-N, risk stratification, Tg_on_, and Tg_off_ before the current course of ^131^I therapy were attributed to non-ER to ^131^I therapy. Among these factors, Tg_on_ and Tg_off_ before the current course of ^131^I therapy were identified as the 2 most important factors that greatly contributed to prediction of ^131^I therapy in RF, as shown in Fig. 3B. Furthermore, we trained 10 RF ensembles of 500 trees each using the influential factors. The frequency of selection of influential factors at the root and the first to third level of trees in the ensembles was calculated to determine their importance. The ranges of the cutoff thresholds used to split the nodes were selected to maximize the impurity gain over all splitting candidates.^20^ One of the RF ensembles of tree is shown in Fig. 3C. A random sample of patients was predicted as ER to ^131^I therapy with this RF ensembles of tree, as shown in Fig. 5D. The prediction was in line with the final follow-up, indicating the effectiveness of the model in predicting treatment response to ^131^I therapy.

### Patient Characteristics for Predicting Treatment Response to TSH Suppression Therapy

At 4-6 months post the last course of ^131^I therapy, Tg_on_ levels in 54.6% (326/597) patients from the training cohort and in 54.8% (141/257) patients from the testing cohort were still more than 0.2 ng/mL. Table 3 shows patients’ characteristics in the training and testing cohort for predicting treatment response to TSH suppression therapy. Except for courses of ^131^I therapy, other factors demonstrated no significant difference between the training and testing cohort.

**Table 3.** Baseline characteristics of differentiated thyroid cancer patients without structural disease in the training and testing cohorts for prediction of treatment response to TSH suppression therapy (N = 467).

Characteristic	Training cohort (*n* = 326)	Testing cohort (*n* = 141)	*P*-value
Sex			.75
Female	218 (66.9%)	92 (65.2%)	
Male	108 (33.1%)	49 (34.8%)	
Age, median (years)	43 (IQR, 35-53)	45 (IQR, 35-54)	.41
Pathology			.18
PTC	321 (98.5%)	136 (96.5%)	
FTC	5 (1.5%)	5 (3.5%)	
Tumor			.55
T1	208 (63.8%)	95 (67.4%)	
T2	65 (19.9%)	21 (14.9%)	
T3	34 (10.4%)	18 (12.8%)	
T4	19 (5.8%)	7 (4.9%)	
Lymph node			.76
N0	22 (6.7%)	8 (5.7%)	
N1a	111 (34.0%)	44 (31.2%)	
N1b	193 (59.3%)	89 (63.1%)	
Stage			.79
I	260 (79.8%)	114 (80.9%)	
II	63 (19.3%)	27 (19.1%)	
III	3 (0.9%)	0 (0%)	
IV	0 (0%)	0 (0%)	
Risk stratification			.90
Low	22 (6.7%)	11 (7.8%)	
Mediate	258 (79.1%)	111 (78.7%)	
High	46 (14.1%)	19 (13.5%)	
RAIU%, median	6.1 (IQR, 3.5-8.5)	6.0 (IQR, 3.7-8.5)	.80
Courses of ^131^I therapy			.08
1	181 (55.5%)	91 (64.5%)	
>1	145 (44.5%)	50 (35.5%)	
TSH_six_, median (mIU/L)	0.12 (IQR, 0.06-0.27)	0.08 (IQR, 0.06-0.23)	.46
Tg_six_, median (ng/mL)	1.08 (IQR, 0.51-1.97)	0.79 (IQR, 0.45-1.86)	.16
TgAb_six_ , median (IU/mL)	12.85 (IQR, 10.00-21.00)	12.00 (IQR, 10.00-18.00)	.45
Thyroid remnant on Rx-WBS of the last course of therapy			.24
Yes	117 (35.9%)	42 (29.8%)	
No	209 (64.1%)	99 (70.2%)	

Abbreviations: TSH, thyroid-stimulating hormone; PTC, papillary thyroid cancer; FTC, follicular thyroid cancer; RAIU, radioiodine uptake; TSH_six_, thyroid-stimulating hormone at 4-6 months after the last course of ^131^I therapy; Tg_six_, stimulated thyroglobulin at 4-6 months after the last course of ^131^I therapy; IQR, interquartile range; Rx-WBS, post-therapeutic whole-body scan.

**Table 4.** Comparison of clinical characteristics of differentiated thyroid cancer patients without structural disease who obtained BR or non-BR to TSH suppression therapy in the training and testing cohorts (*N* = 467).

Characteristic	Training cohort (*n* = 326)			Testing cohort (*n* = 141)		
	BR (*n* = 155)	Non-BR (*n* = 171)	*P*-value	BR (*n* = 72)	Non-BR (*n* = 69)	*P*-value
Sex			.01			.01
Female	121 (78.1%)	97 (56.7%)		60 (83.3%)	32 (46.4%)	
Male	34 (21.9%)	74 (43.3%)		12 (16.7%)	37 (53.6%)	
Age, median (years)	45 (IQR, 35-54)	43 (IQR, 34-53)	.38	46 (IQR, 38-55)	42 (IQR, 35-54)	.16
Pathology			1.00			1.00
PTC	153 (98.7%)	168 (98.2%)		69 (95.8%)	67 (97.1%)	
FTC	2 (1.3%)	3 (1.8%)		3 (4.2%)	2 (2.9%)	
Tumor			.88			.49
T1	96 (61.9%)	112 (65.5%)		45 (62.5%)	50 (72.5%)	
T2	32 (20.7%)	33 (19.3%)		11 (15.3%)	10 (14.5%)	
T3	18 (11.6%)	16 (9.4%)		12 (16.7%)	6 (8.7%)	
T4	9 (5.8%)	10 (5.8%)		4 (5.5%)	3 (4.3%)	
Lymph node			.22			.01
N0	9 (5.8%)	13 (7.6%)		8 (11.1%)	0 (0%)	
N1a	60 (38.7%)	51 (29.8%)		26 (36.1%)	18 (26.1%)	
N1b	86 (55.5%)	107 (62.6%)		38 (52.8%)	51 (73.9%)	
Stage			.52			.83
I	120 (77.4%)	140 (81.9%)		59 (81.9%)	55 (79.7%)	
II	33 (21.3%)	30 (17.5%)		13 (18.1%)	14 (20.3%)	
III	2 (1.3%)	1 (0.6%)		0 (0%)	0 (0%)	
IV	0 (0%)	0 (0%)		0 (0%)	0 (0%)	
Risk stratification			.01			.01
Low	10 (6.5%)	12 (7.0%)		10 (13.8%)	1 (1.4%)	
Mediate	133 (85.8%)	125 (73.1%)		57 (79.2%)	54 (78.3%)	
High	12 (7.7%)	34 (19.9%)		5 (7.0%)	14 (20.3%)	
RAIU%, median	6.0 (IQR, 4.0-8.3)	6.2 (IQR, 3.2-8.9)	.78	5.5 (IQR, 3.7-8.6)	6.2 (IQR, 3.7-8.4)	.88
Courses of ^131^I therapy						.01
1	115 (74.2%)	66 (38.6%)		58 (80.6%)	33 (47.8%)	
>1	40 (25.8%)	105 (61.4%)		14 (19.4%)	36 (52.2%)	
TSH_six_, median (mIU/L)	0.15 (IQR, 0.06-0.4)	0.08 (IQR, 0.06-0.21)	.01	0.13 (IQR, 0.07-0.33)	0.08 (IQR, 0.06-0.163)	.03
Tg_six_, median (ng/mL)	0.65 (IQR, 0.38-1.34)	1.65 (IQR, 0.76-2.65)	.01	0.36 (IQR, 0.35-1.07)	1.47 (IQR,0.62-3.57)	.01
TgAb_six_, median (IU/mL)	14.00 (IQR, 10.00-23.00)	12.00 (IQR, 10.00-16.80)	.36	12.25 (IQR, 10.00-20.33)	12.00 (IQR, 10.00-18.00)	.71
Thyroid remnant on Rx-WBS of the last course of ^131^I therapy			.01			.01
Yes	16 (10.3%)	101 (59.1%)		58 (80.6%)	33 (47.8%)	
No	139 (89.7%)	70 (40.9%)		14 (19.4%)	36 (52.2%)	

Abbreviations: TSH, thyroid-stimulating hormone; BR, biochemical remission; PTC, papillary thyroid cancer; FTC, follicular thyroid cancer; RAIU, radioiodine uptake; TSH_six_, thyroid-stimulating hormone at 4-6 months after the last course of ^131^I therapy; Tg_six_, stimulated thyroglobulin at 4-6 months after the last course of ^131^I therapy; TgAb_six_, antithyroglobulin antibody at 4-6 months after the last course of ^131^I therapy; IQR, interquartile range; Rx-WBS, post-therapeutic whole-body scan.

### Predicting Treatment Response to TSH Suppression Therapy

As shown in Table 4, by evaluating ∆Tg_on_% within 6 months (from 6 months after the last course of ^131^I therapy to 12-24 months after the last course of ^131^I therapy), 47.5% (15?5?/326) patients received BR to TSH suppression therapy, and 52.5% (171/326) patients obtained non-BR to TSH suppression therapy in the training cohort, while 51.1% (72/141) patients received BR to TSH suppression therapy and 48.9% (69/141) patients obtained non-BR to TSH suppression therapy in the testing cohort.

**Figure 4.**
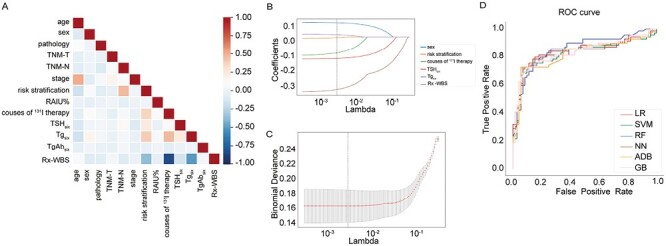
Establishment and validation of machine learning model for predicting treatment response to thyrotropin (TSH) suppression therapy in differentiated thyroid cancer patients without structural disease. (**A**) Internal correlation between the enrolled factors associated with TSH suppression therapy. (**B**) The least absolute shrinkage and selection operator for influential factors that associated with treatment response to TSH suppression therapy. (**C**) The optimal value of λ at the minimum mean square error for feature selection. (**D**) Area under the receiver operating characteristic curve for predicting treatment response to TSH suppression therapy.

 There was no high internal correlation between the factors, as shown in Fig. 4A. Several variables including sex, risk stratification, RAIU% before the last course of ^131^I therapy, TSH_six_, Tg_six_, courses of ^131^I therapy, thyroid remnant on Rx-WBS of the last course of ^131^I therapy showed significant difference in DTC patients with and without BR to TSH suppression therapy after initial screening. After LASSO regression with cross-testing, sex, risk stratification, TSH_six_, Tg_six_, courses of ^131^I therapy, thyroid remnant on Rx-WBS of the last course of ^131^I therapy were selected as the best predictors for the probability of non-BR to TSH suppression therapy, as shown in Fig. 4B-C.

LG, SVM, RF, NN, ADA, and GB were used to build multiple models during the training process, and the performance of each algorithm was evaluated by AUC in the testing cohort. RF model had the best performance with the highest accuracy of 78.7% and AUC of 0.857, as shown in Fig. 4D and Supplementary Table S2.

**Figure 5.**
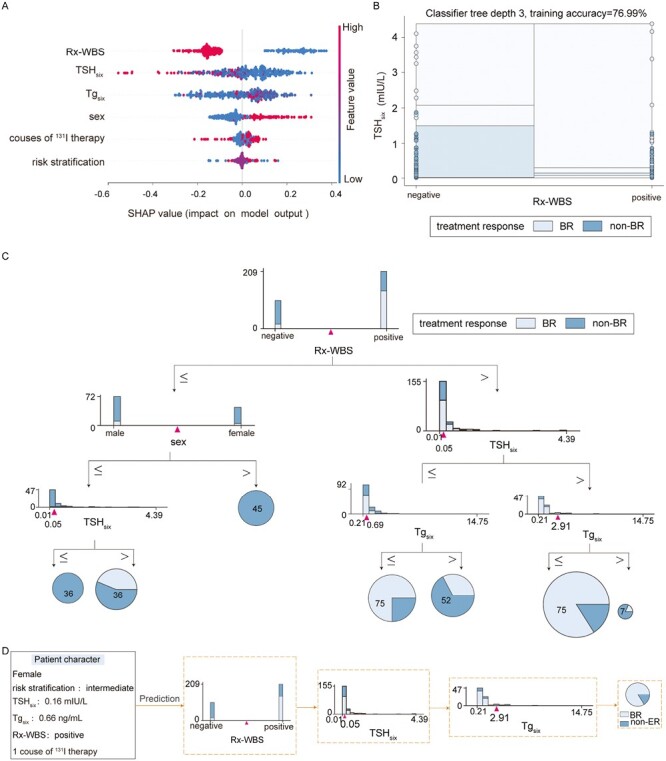
Random forest (RF) model for predicting treatment response to thyrotropin (TSH) suppression therapy in differentiated thyroid cancer patients without structural disease. (**A**) SHapley Additive exPlanations summary plot for influential factors. (**B**) Correlation between TSH and remnant thyroid available on post-therapeutic whole-body scan in RF. (**C**) One of the RF ensembles of tree for predicting treatment response to TSH suppression therapy. (**D**) A random sample of a patient using RF to predict treatment response to TSH suppression therapy.

Figure 5A illustrates the influential factors in RF by SHAP analysis. Visible thyroid remnant on Rx-WBS of the last course of ^131^I therapy and higher TSH_six_ were positively associated with BR to TSH suppression therapy, and the above indicators were identified as the most 2 important factors that greatly contributed to the prediction of TSH suppression therapy in RF, as shown in Fig. 5B. One of the RF ensembles of tree is shown in Fig. 5C. A random sample of patients was predicted as BR to TSH suppression therapy with this RF ensembles of tree, as shown in Figure 5D. The prediction was in line with the final follow-up, indicating the effectiveness of the model in predicting treatment response to TSH suppression therapy.

## Discussion

To our knowledge, this is the first report on developing machine learning models for predicting treatment response to ^131^I therapy and TSH suppression therapy. Different machine learning models for screening the patients who benefit from ^131^I therapy and TSH suppression therapy were constructed based on pre-treatment clinical variables and biochemical markers in a large cohort. The best predictive performance was achieved by RF. These innovative models showed favorable predictive accuracy for predicting ER to ^131^I therapy and BR to TSH suppression therapy, which can aid clinicians in making informed decisions about patient care.

^131^I therapy has been used in clinical treatment of thyroid disorder for 80 years.^21^ Overwhelmingly, ^131^I therapy has been guided by several factors such as information derived from surgical histopathology, radioiodine scintigraphy, and TSH or Tg levels in previous studies.^22,23^ In our study, sex, risk stratification, TNM-N, RAIU%, Tg_on_, Tg_off_, TSH_off_, and courses of ^131^I therapy before the current course of ^131^I therapy, were all identified as significant factors for predicting treatment response to ^131^I therapy. We found that male gender demonstrated better Tg remission, which is consistent with previous research suggesting that higher levels of estrogen may increase Tg levels. This may due to the fact that estrogens increase the secretion of mutagenic molecules in the thyroid cell and favor the proliferation of tumoral cells.^24^ Additionally, we found that ER is slightly correlated with risk stratification. ^131^I therapy is generally recommended for patients with high-risk features.^25^ However, patients with high-risk stratification are difficult to get ER to ^131^I therapy, as it indicates that patients with high-risk stratification may require different treatment strategies to control Tg level. Moreover, thyroid remnant is the major contribution of high Tg even after the total thyroidectomy. RAIU% is carried out to test remnant thyroid function before ^131^I therapy.^26^ Detectable Tg before the current course of ^131^I therapy also indicates thyroid remnant or biochemical disease for this group of patients, which can help to guide treatment decisions.^12^ Li et al showed that Tg_off_ was closely associated with ER in initial ^131^I therapy. The cutoff value of Tg_off_ for predicting ER was 6.915 ug/L, with a sensitivity of 69.2% and a specificity of 89.4%.^27^ In our study, we also found that both RAIU% and Tg had great impact on the prediction of ER to ^131^I therapy. Almost all patients demonstrated ^131^I uptake in the thyroid gland bed or thyrolingual cyst on Rx-WBS after ^131^I therapy, especially after the initial course of ^131^I therapy. Tg decrease was available for these individuals with increasing number of ^131^I therapies, indicating that repeated courses of ^131^I therapy can be effective in achieving treatment response.

Patients with high Tg level and negative findings on Rx-WBS will likely need additional evaluations and possibly even complementary therapies according to previous studies.^3,12,28^ Interestingly, Tg_on_ in 26.3% (49/186) patients in the training cohort and 26.6% (21/79) patients in the testing cohort dropped to less than 0.2 ng/mL after additional courses of ^131^I therapy, despite the absence of ^131^I-avid metastatic lesions on after the current course of ^131^I therapy. This suggests that patients with high Tg levels are indicators for adjuvant therapy, which can decrease Tg level and facilitate initial staging and follow-up.^28,29^

TSH suppression therapy is a therapeutic intervention in thyroid cancer after thyroidectomy and ^131^I therapy that can slow the growth of thyroid cancer cells and lead to Tg decline by maintaining subnormal TSH levels.^30^ Of the clinical and biochemical factors selected in the present study, our study presented initial solid proof that the evidence of thyroid remnant on Rx-WBS of the last course of ^131^I therapy and high TSH clearly identify those patients who may benefit from TSH suppression therapy. High levels of TSH indicates insufficient dosage of LT4. On the assumption that sufficient dosage of LT4 were given, Tg levels would decline rapidly. Patients with intermediate- and high-risk DTC might not benefit from TSH suppression therapy.^31^ Thyroid remnant on Rx-WBS at the last course of ^131^I therapy indicated that remnant thyroid may be due to Tg level. To avoid interference of ^131^I therapy on Tg_on_ level, the treatment response to TSH suppression therapy was assessed by comparing Tg_on_ level at 6 months post the last course of ^131^I therapy with Tg_on_ level at 12-24 months post the last course of ^131^I therapy. However, we can’t be sure that ^131^I therapy had no effect on Tg_on_ at 6 months post the last course of ^131^I therapy. Additionally, the percentage of patients who achieve nadir Tg continues to increase over time under TSH suppression, and that it may be reasonable to resist giving additional therapies for patients with persistently low Tg level without the structural evidence of disease.^32^ In the present study, we also observed Tg decline in patients with relatively low level of Tg at the initial time. However, almost half of patients in our study who have relatively high level of Tg did not obtain Tg decrease. Poor adherence to medication regimens, or other factors that affect the absorption or metabolism of the medication is one possible explanation for these patients.^33^ Another possible explanation is invalid ^131^I therapy. ^131^I therapy is typically used to eliminate any remaining thyroid cancer cells after surgery, but it may not be effective in all cases, particularly in patients with more advanced or aggressive disease. In some cases, a rising Tg level may be an early indicator of recurrence or progression of the disease, and further testing or imaging studies may be necessary to confirm the diagnosis. Moreover, simple clinical parameters, such as sex has proved to be associated with Tg spontaneous decline; however, as they are not included in the ATA system. Similar with the findings in prediction of treatment response to ^131^I therapy, male gender is more sensitive to TSH suppression therapy. However, it is important to note that the selection of treatment approaches should always be individualized based on each patient’s specific circumstances and needs. The use of TSH suppression therapy should be carefully weighed against the potential risks and benefits for each patient.

In this study, we combined the influential factors to predict the treatment response to ^131^I therapy or TSH suppression therapy with 6 different machine learning models, including LR, SVC, RF, NN, ADA, and GB. These models demonstrated comparable performance in predicting treatment response, in which RF models yield the highest accuracy and AUC. The RF algorithm is an ensemble of decision trees based on the bagging and random subspace concepts. In an RF model, multiple decision trees are created using a random subset of the available features. Each tree is trained on a different subset of the data, and the resulting predictions from all of the trees are combined to produce a final prediction. The randomization of the data subsets helps to improve the generalization and to reduce overfitting of the final model. Additionally, the combination of multiple decision trees helps to capture complex relationships between the features and the target variables, yielding high accuracy of the model.^34,35^ RF models integrating pre-treatment clinical and biochemical findings before ^131^I therapy and TSH suppression therapy can better distinguish the patients who may benefit from therapy before intervention.

Some limitations existed in this study. Firstly, our study was conducted retrospectively and enrolled patients with DTC without structural disease who received at least one course of ^131^I therapy, but this study did not represent the cohort that did not receive ^131^I therapy in our institute; the inclusion of patients might have been influenced by selection bias. Secondly, all patients came from a single institute. External testing outside our institute would be more forceful. Thirdly, the factors for predicting treatment response were derived from the pre-treatment clinical variables or biochemical markers. However, some important information that might be associated with treatment response of ^131^I therapy, such as BRAF status, were not included in this study due to too much missing data.

## Conclusion

This study demonstrates that machine-learning models, especially the RF algorithm, are useful tools that may predict treatment response to ^131^I therapy and TSH suppression therapy in patients with DTC but without structural disease based on pre-treatment routine clinical variables and biochemical markers.

## Supplementary Material

Supplementary material is available at *The Oncologist* online.

oyad252_suppl_Supplementary_Material

## Data Availability

All datasets in the current study are available from the corresponding author on reasonable request.
