# Supplementary material for: Random Forest for Predicting Treatment Response to Radioiodine and Thyrotropin Suppression Therapy in Patients With Differentiated Thyroid Cancer But Without Structural Disease
Source: Oncologist. 2023 Sep 5;29(1):e68–80. doi: 10.1093/oncolo/oyad252 (PMC10769791; doi:10.1093/oncolo/oyad252)
Supplement: oyad252_suppl_Supplementary_Material [file oyad252_suppl_supplementary_material.zip › Supplementary table 1.docx]

**Supplementary table 1.** **Prediction treatment response to ^131^I therapy with different models in the testing cohort.**

|  | AUC | Accuracy | Sensitivity | Specificity |
| --- | --- | --- | --- | --- |
| LR | 0.878 | 81.3% | 74.3% | 89.7% |
| SVC | 0.879 | 80.2% | 76.4% | 84.6% |
| RF | 0.896 | 81.3% | 79.5% | 82.9% |
| NN | 0.828 | 74.3% | 75.0% | 73.5% |
| ADA | 0.868 | 79.0% | 74.4% | 82.9% |
| GB | 0.886 | 78.2% | 74.4% | 81.4% |

LR, Logistic Regression; SVC, Support Vector Machine; RF, Random Forest; NN, Neural Networks; ADA, Adaptive Boosting; GB, Gradient Boost.
