# Supplementary material for: Random Forest for Predicting Treatment Response to Radioiodine and Thyrotropin Suppression Therapy in Patients With Differentiated Thyroid Cancer But Without Structural Disease
Source: Oncologist. 2023 Sep 5;29(1):e68–80. doi: 10.1093/oncolo/oyad252 (PMC10769791; doi:10.1093/oncolo/oyad252)
Supplement: oyad252_suppl_Supplementary_Material [file oyad252_suppl_supplementary_material.zip › Supplementary table 2.docx]

**Supplementary table 2. Prediction treatment response to TSH suppression therapy with different models in the testing cohort.**

|  | AUC | Accuracy | Sensitivity | Specificity |
| --- | --- | --- | --- | --- |
| LR | 0.841 | 81.6% | 73.9% | 88.9% |
| SVC | 0.821 | 79.4% | 72.5% | 86.1% |
| RF | 0.857 | 78.7% | 79.7% | 77.8% |
| NN | 0.827 | 80.9% | 73.9% | 87.5% |
| ADA | 0.819 | 80.9% | 72.5% | 88.9% |
| GB | 0.832 | 78.0% | 76.8% | 79.2% |

LR, Logistic Regression; SVC, Support Vector Machine; RF, Random Forest; NN, Neural Networks; ADA, Adaptive Boosting; GB, Gradient Boost.
